# A molecular density functional theory approach to electron transfer reactions

**DOI:** 10.1039/c8sc04512g

**Published:** 2018-12-12

**Authors:** Guillaume Jeanmairet, Benjamin Rotenberg, Maximilien Levesque, Daniel Borgis, Mathieu Salanne

**Affiliations:** a Sorbonne Université , CNRS , Physico-Chimie des Électrolytes et Nanosystèmes Interfaciaux , PHENIX , F-75005 Paris , France . Email: guillaume.jeanmairet@sorbonne-universite.fr; b Réseau sur le Stockage Électrochimique de l'Énergie (RS2E) , FR CNRS 3459 , 80039 Amiens Cedex , France; c PASTEUR , Département de chimie , École Normale Supérieure , PSL University , Sorbonne Université , CNRS , 75005 Paris , France; d Maison de la Simulation , CEA , CNRS , Université Paris-Sud , UVSQ , Université Paris-Saclay , 91191 Gif-sur-Yvette , France

## Abstract

Beyond the dielectric continuum description initiated by Marcus theory, the standard theoretical approach to study electron transfer (ET) reactions in solution or at interfaces is to use classical force field or *ab initio* molecular dynamics simulations. We present here an alternative method based on liquid-state theory, namely molecular density functional theory, which is numerically much more efficient than simulations while still retaining the molecular nature of the solvent. We begin by reformulating molecular ET theory in a density functional language and show how to compute the various observables characterizing ET reactions from an ensemble of density functional minimizations. In particular, we define within that formulation the relevant order parameter of the reaction, the so-called vertical energy gap, and determine the Marcus free energy curves of both reactant and product states along that coordinate. Important thermodynamic quantities such as the reaction free energy and the reorganization free energies follow. We assess the validity of the method by studying the model Cl^0^ → Cl^+^ and Cl^0^ → Cl^–^ ET reactions in bulk water for which molecular dynamics results are available. The anionic case is found to violate the standard Marcus theory. Finally, we take advantage of the computational efficiency of the method to study the influence of a solid–solvent interface on the ET, by investigating the evolution of the reorganization free energy of the Cl^0^ → Cl^+^ reaction when the atom approaches an atomistically resolved wall.

## Abstract 2

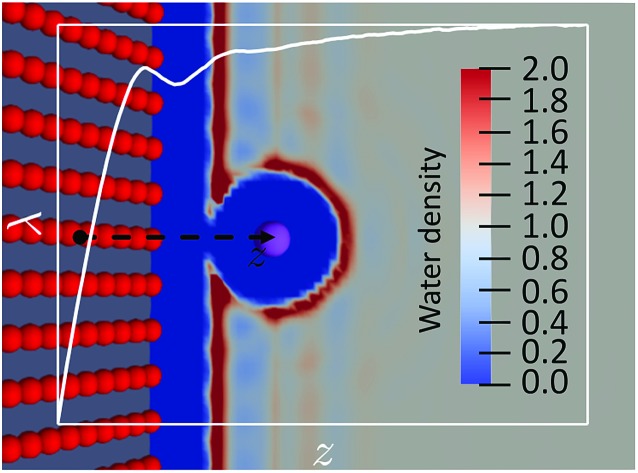
Molecular density functional theory, an efficient computational tool, provides new insights into the study of electron transfer reactions in bulk and interfacial water.

## 

Electron transfer (ET) reactions play a central role in a wide range of chemical systems, including energy storage and harvesting in electrochemical devices or biological processes such as aerobic respiration and photosynthesis. This ubiquity can explain the considerable amount of experimental, theoretical and simulation studies that have been devoted to this class of reactions.^1^ The widely accepted theory of ET reaction in solution was proposed by Marcus.^2^–^4^ It is based on the description of the solvent by a dielectric continuum. The macroscopic fluctuations of the solvent are represented by an out-of-equilibrium polarization field, and the free energy is a functional depending quadratically on this polarization. It eventually provides a simple two-state picture, where the free energy of each state depends quadratically on a reaction coordinate. This famous two-parabola picture has been used with great success to interpret experimental results and to make predictions.^5^ However, Marcus theory does not take into account the molecular nature of the solvent which can break the linear assumption of solvent response. In such cases, we must resort to molecular simulation.

The vast majority of simulation studies on ET reaction have been carried out using Molecular Dynamics (MD). For example, the pioneering work of Warshel demonstrated that the vertical energy gap is the appropriate reaction coordinate^6^ and that the fluctuations of this quantity are Gaussian. Such Gaussian statistics give rise to the famous parabola picture of Marcus for the free energy profile. A strict Gaussian behaviour is equivalent to a linear response of the solvent to the field generated by the solute; it also implies that the two free energy parabolas have the same curvature because the solvent fluctuations are identical for the two states.^7^,^8^

This validity of the Gaussian assumption has been verified in numerous studies since, for ET in solution^9^,^10^ or in complex biological systems,^11^,^12^ using either classical or *ab initio* MD. However, there is evidence that some systems do not obey the Marcus assumption *i.e.* the free energy curves of the two states cannot be represented by a pair of identical parabolas. There are several possible origins of such a discrepancy,^13^ in particular the fact that reactant and product may have quite different solvation states. This can happen when the ET occurs between neutral and charged states, as predicted by Kakitani and Mataga^14^–^16^ and observed since in classical^17^–^19^ and *ab initio*^20^,^21^ simulations. Several extensions of Marcus theory have been put forward to take into account the various origins of non-linearity.^21^–^24^

The investigation of ET reactions by MD is quite challenging since it usually requires the computation of the free energy curves as a function of an order parameter which remains a demanding task. Indeed, it necessitates a proper sampling of the solvent configurations around the barrier. If the activation energy is high it requires the use of biases such as umbrella sampling^25^ coupled with histogram analysis techniques to reconstruct the unbiased data.^26^–^28^ This typically implies to run simulations on half a dozen fictitious intermediate states to study a single system.

To compute free energies, there exist alternative techniques based on statistical theory of liquids, which offer the advantage of keeping a molecular description of the solvent while avoiding to sample the instantaneous microscopic degrees of freedom. Among the different approaches one can mention integral equation theory either in its molecular^29^ or multiple sites formulation (RISM)^30^,^31^ and its 3D-RISM version.^32^,^33^ Another method is the classical density functional theory (cDFT) of liquids^34^,^35^ which describes the response of a fluid in the presence of a perturbation by introducing a functional of the fluid density. Minimization of the functional yields the grand potential at equilibrium fluid density. Some of us have previously introduced the molecular density functional theory (MDFT)^36^,^37^ which is able to provide the solvation free energy and the solvation structure of any solute embedded in a molecular solvent described by its inhomogeneous density field. The solvent density is a function of space coordinates and of the orientation; hence the functional must be minimized on a 6D grid: 3 dimensions for the cartesian coordinates and 3 dimensions for the three Euler angles. This formalism can be used to solvate any simple or complex solutes.^38^ We proposed functionals for several solvents^39^,^40^ with particular attention paid to the case of water.^41^,^42^ The most advanced version of the functional is equivalent to the molecular Ornstein–Zernike theory supplemented by the hypernetted-chain closure (HNC)^43^ for the solute–solvent correlations and can be minimized efficiently thanks to the use of rotational invariants in an optimal frame. The accuracy on the predictions of solvation free energies is promising as illustrated on the FreeSolv database.^44^ We shall take advantage of this accuracy to put forward an efficient way to compute the free energy curves.

An application of the MDFT formalism to ET reactions in acetonitrile was proposed some years ago.^39^ In this article we extend this approach and apply it to ET in aqueous solutions. In Section 1, after recalling some basics of ET theory and giving a very short description of the MDFT framework, we show how to compute the key quantities of ET reactions with MDFT. In particular, we show that the average vertical energy gap is an appropriate order parameter for the ET reaction. We prove that for a given set of external potentials the free energy functional is actually a function of this order parameter. We derive expressions to compute the free energy curves (FEC) and the reorganization free energies.

In Section 2 we first validate the framework on the simplest solute in water, *i.e.* a single neutral or charged chlorine atom modeled by a Lennard-Jones site, before studying the influence of the presence of a solid-solvent interface on the reorganization free energy; to this purpose we investigate the ET of this solute as a function of its distance to an atomistically resolved wall.

## 1 Theory

### 1.1 Electron transfer reaction

**Fig. 1:**
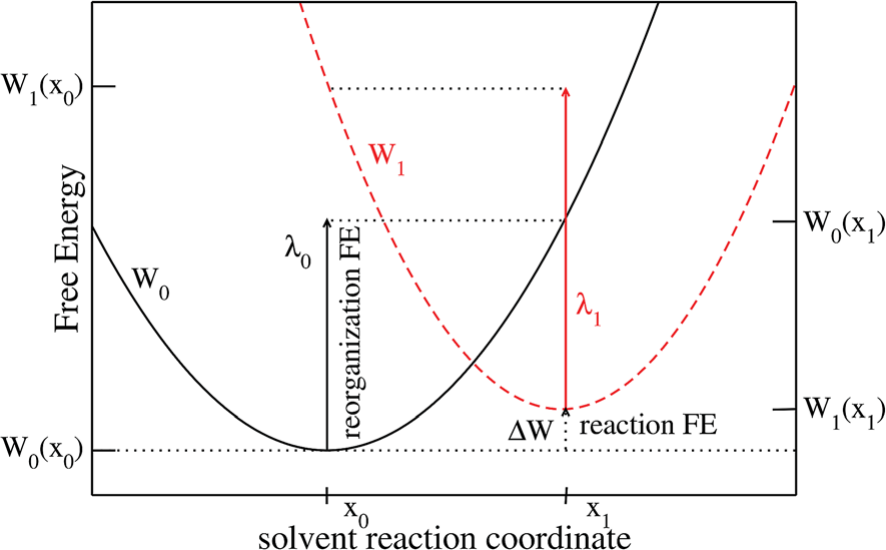
Schematic representation of a solvent controlled ET reaction. The diabatic free energy curves for state 0 and 1 are represented in solid black and dashed red, respectively. The reorganization free energies *λ*_0_ and *λ*_1_ for the two states are represented with solid arrows, the reaction free energy with a dotted arrow.

We limit ourselves to the study of ET reactions of solutes which interact with the solvent through a classical force field. Moreover, the solutes we consider in this article are rigid entities composed of a set of Lennard-Jones sites and point charges. An ET reaction involving two solutes of this type would correspond to an outer-sphere ET because there are no structural changes of the solutes. This implies that the ET reaction is completely controlled by the solvent response, as considered in Marcus' original paper.^3^ The physics of the system can be described by the two crossing free energy curves of the system before (0) and after (1) the ET. A schematic view of the two FEC is presented in Fig. 1 where some of the quantities necessary to describe the process are shown. The order parameter *x* describes the solvent configuration around the solute, thus the abscissa *x*_0_ of the minimum of the FEC *W*_0_ corresponds to a solvent in equilibrium with state 0. We emphasize that several microscopic solvent configurations correspond to an identical value of the order parameter.

Values of the order parameter differing from *x*_0_ correspond to solvent configurations that are not in equilibrium with state 0. The more the solvent configuration differs from equilibrium, the more the free energy increases. The difference between the minima of the 2 FEC corresponds to the free energy difference between the two states, each surrounded by a solvent in equilibrium with these states, *i.e.* the reaction free energy, Δ*W*. Two others key quantities appear in Fig. 1: the reorganization free energies *λ*_0_ (resp. *λ*_1_) which represent the cost in free energy to solvate state 0 (resp. 1) in a solvent in equilibrium with the other state. The difference in free energy between the transition state (the crossing point) and state 0 controls the kinetics of the 0 → 1 reaction.

We emphasize that in the Marcus picture, the solvent is treated as a continuum which responds linearly to the electric field generated by the solute. This implies that the FEC of the 2 states are identical parabolas. As a consequence, there is a unique reorganization free energy parameter *λ* = *λ*_0_ = *λ*_1_. The objective of the present paper is to show how to compute the various quantities appearing in Fig. 1, within MDFT. This could be a way to test the validity of Marcus assumption when the molecular nature of the solvent is taken into account, while taking advantage of the numerical efficiency of MDFT compared to MD.

### 1.2 Molecular density functional theory

We briefly recall the fundamentals of MDFT which belong to the more general class of cDFT. Based on the Hohenberg–Kohn ansatz,^45^ Mermin introduced the framework of density functional theory (DFT) at finite temperature for the inhomogeneous electron gas.^34^ Later, Evans rewrote the theory for a classical system, setting the foundations of cDFT which describes the response of a fluid to an external perturbation.^35^

MDFT is designed to study solvation problems. The fluid perturbed by the presence of one or several solutes is described by its density field. In the following we will always consider liquids, referred to as the solvent. It is advantageous to define a new functional *F*, as the difference between the grand potential functional of the solvent in the presence of the solute *Θ* and that of the homogeneous solvent,

1 *F*[*ρ*] = *Θ*[*ρ*] – *Θ*_H_.

With this definition, the functional at its minimum is equal to the solvation free energy. Because both the solute and the solvent are in most cases molecules with several atomic sites, their interactions depend on both the position of the centres of mass (CM) and orientations. Hence, the solvent density will be denoted by *ρ*(***r***,**Ω**) where ***r*** is the position in cartesian coordinates and **Ω** the orientation described by three Euler angles (*θ*,*φ*,*ψ*). The density minimizing the functional is the full equilibrium solvent density around the solute which may be integrated to recover the usual radial distribution functions.

The usual strategy to have a workable expression of this functional is to split it into the sum of ideal, excess and external contributions,

2 *F*[*ρ*] = *F*_id_[*ρ*] + *F*_ext_[*ρ*] + *F*exactexc[*ρ*].

In eqn (2) the ideal term corresponds to the entropy of the non-interacting fluid, which reads3
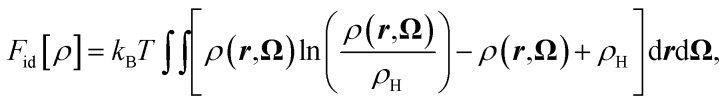
where *k*_B_ is the Boltzmann constant, *T* the temperature in Kelvin and 
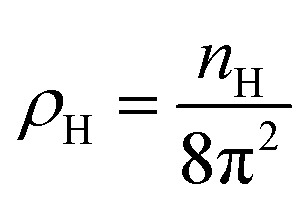
 with *n*_H_ the density of the homogeneous solvent. The second term in eqn (2) account for the perturbation by the solute. The solute acts on the solvent *via* an ”external” potential *V*_ext_, typically the sum of a Lennard-Jones term and of an electrostatic term. In the usual case of pair-wise additive interaction, *V*_ext_ reads4
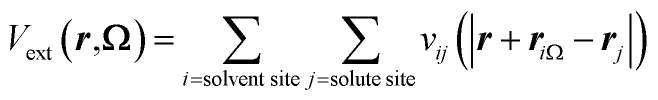
where *v*_*ij*_ is the pair potential between site *i* of solvent and site *j* of the solute and ***r***_*i***Ω**_ denotes the position of site *i* when the solvent molecule has the orientation **Ω**. The expression of the external functional is:5
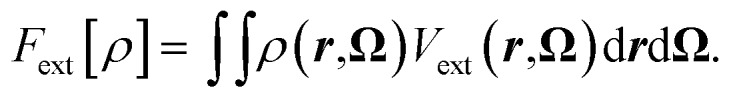



Finally, the last term corresponds to the solvent–solvent interactions. An exact expression for this term is available^46^ but in practice it is rewritten as the sum of two terms

6 *F*exactexc[*ρ*] = *F*_exc_[*ρ*] + *F*_b_[*ρ*],

where *F*_exc_ is an approximation of the excess functional. This defines the correction or ”bridge” functional *F*_b_ as the difference between the exact functional and this approximation. To date, the most advanced expression of *F*_exc_ for water is that recently used by Ding and coworkers which corresponds to the hypernetted chain approximation.^43^7
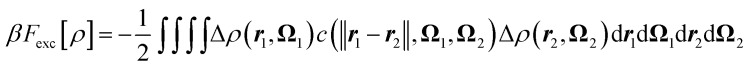
where *β* = (*k*_B_*T*)^–1^ and *c*(∥***r***_1_ – ***r***_2_∥,**Ω**_1_,**Ω**_2_) is the bulk direct correlation function while Δ*ρ* = *ρ* – *ρ*_H_. *c* depends on the distance between 2 solvent molecules and on their relative orientation defined by six Euler angles instead of 4 in the case of acetonitrile.^39^ The calculation of the functional of eqn (7) in this general case is still feasible thanks to the efficient FFT algorithm^47^ to handle the spatial convolution and to the use of rotational invariants to handle the angular one.^48^–^50^

We have previously developed several approximations for the bridge term *F*_b_^51^–^53^ but we shall neglect it in this paper and refer to this functional formulation as the HNC functional. While the numerical problem involved in MD is the sampling of phase space, MDFT involves an optimization which is numerically more efficient. Consequently, to compute solvation free energies of a spherical solute in water, MDFT requires ≈10 cpu minutes with our lab-developed program while it requires ≈100 cpu hours to compute the same quantity with commercially available MD codes.

### 1.3 ET reaction in the MDFT framework

#### 1.3.1 Theory

From a MDFT perspective, the two states 0 and 1 of the ET reaction correspond to two functionals *F*_0_ and *F*_1_ differing only by their external potentials *V*_0_ and *V*_1_ in eqn (5). If we denote by *ρ*_0_ and *ρ*_1_ the equilibrium solvent densities of states 0 and 1, obtained by minimization of *F*_0_ and *F*_1_, the reaction free energy can be expressed as

8 Δ*W* = *F*_1_[*ρ*_1_] – *F*_0_[*ρ*_0_] + Δ*E*_0_.

The first two terms represent the solvent contribution to the free energy while Δ*E*_0_ is the difference in energy between the 2 solutes in vacuum. In this paper we restrict the study to rigid classical solutes with no intramolecular potentials, so that last term vanishes.

In his original work, Marcus estimated the free energy cost to solvate a solute within a solvent where polarization is not in equilibrium with the electric field generated by the solute. In the MDFT framework the density field contains all the structural equilibrium information of the solvent, including its polarization. We can consider MDFT as a more general field theory than that used by Marcus. Nevertheless, the density field itself remains a complicated object. To facilitate our understanding it is useful to define a solvent reaction coordinate *i.e.* a scalar quantity which is uniquely defined by the density field. By introducing a class of intermediate potentials interpolating between state 0 and state 1, we show that the average vertical energy gap is an appropriate order parameter. We then derive an expression for the free energies of states 0 and 1 as functions of the average vertical energy gap.

States 0 and 1 are characterized by the following Hamiltonian

9 *H*_*η*_ = *K* + *U* + *V*_*η*_.

where *η* = 0 or 1, *K* is the kinetic energy and *U* is the potential energy. For each state, the equilibrium probability distribution in the Grand Canonical ensemble is

10 *f*_*η*_(***X***^*N*^,***p***^*N*^) = Ξ_*η*_^–1^exp[–*β*(*H*_*η*_(***X***^*N*^,***p***^*N*^) – *μN*)],

where *μ* is the chemical potential of the solvent and (***X***^*N*^,***p***^*N*^) is a point in phase space ***X*** denotes the couple (***r***,**Ω**) describing the CM and orientation of a solvent molecule with momentum ***p***. Ξ_*η*_ is the corresponding grand partition function:

11 Ξ_*η*_ = Tr[exp(–*β*(*H*_*η*_ – *μN*))],

where Tr denotes the classical trace12
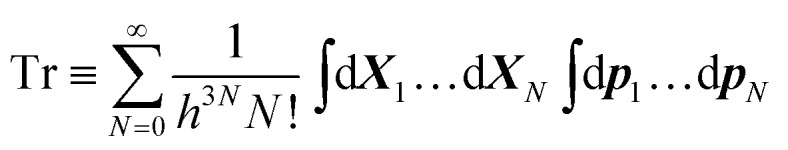
and *h* is the Planck constant. We now introduce a class of external potentials defined as linear combinations of *V*_0_ and *V*_1_

13 *V*_*η*_ = *V*_0_ + *η*(*V*_1_ – *V*_0_) with *η* ∈ [0,1].

This defines the corresponding set of Hamiltonians (eqn (9)), probability distributions (eqn (10)) and grand partition functions (eqn (11)) for any value of *η*. Since for physically relevant cases *V*_0_ and *V*_1_ differ by more than a constant, any value of *η* defines a unique potential *V*_*η*_ (up to an irrelevant constant). Because of this uniqueness of the potential, a unique equilibrium solvent density *ρ*_*η*_ is associated with any value of *η*. This is a consequence of the cDFT principle^35^ which implies a one-to-one mapping between external potential, equilibrium distribution and equilibrium solvent density.†
†Note that this is true for any *V*_*η*_ = *V*_0_ + *s*(*η*) (*V*_1_ – *V*_0_), as long as *s* is a strictly increasing continuous function with *s*(0) = 0 and *s*(1) = 1.


We define the average vertical energy gap, related to an equilibrium density *ρ*_*η*_ by14
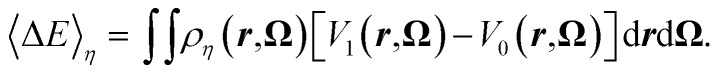



This quantity represents the energy difference between states 1 and 0 solvated in the solvent of density *ρ*_*η*_. We prove in Appendix A that, for the family of potentials in eqn (13), , 〈ΔΔ*E*〉_*η*_ is an adequate order parameter since it uniquely defines *ρ*_*η*_. Thus, the free energy of any state is a function of . Thus, the free energy of any state is a function of 〈ΔΔ*E*〉_*η*_. For instance, for state 0 it reduces to

15 *F*_0_((〈ΔΔ*E*〉_*η*_) ≡ *F*_0_[*ρ*_*η*_].

Note that the average free energy gap defined in eqn (14) differs from the microscopic version, Δ*E*, used in MD;

16 Δ*E*({***R***}) = *E*_1_({***R***}) – *E*_0_({***R***}),

with {***R***} denoting the whole set of coordinates of solvent molecules, but they are actually related by

17 〈ΔΔ*E*({***R***})})〉_*η*_ = = 〈ΔΔ*E*〉_*η*_,

where on the left hand side where on the left hand side 〈..〉..where on the left hand side 〈..〉_*η*_ denotes the thermodynamic average on the potential energy surface *η*. . 〈ΔΔ*E*〉_*η*_ is also frequently reported in MD studies of ET since it is another measure of the validity of Marcus theory which predicts that it varies linearly with the coupling parameter. Our approach is closer to Marcus' original work^54^ where he mentioned that the “equivalent equilibrium distribution would be obtained in a corresponding equilibrium system in which the charges on the two central ions” are linear combinations of the original ones.

We can now express the reorganization free energies displayed in Fig. 1 as

18 *λ*_0_ = *F*_0_((〈ΔΔ*E*〉_1_) – *F*_0_((〈ΔΔ*E*〉_0_) = *F*_0_[*ρ*_1_] – *F*_0_[*ρ*_0_] = Δ*W* – − 〈ΔΔ*E*〉_1_,

19 *λ*_1_ = *F*_1_((〈ΔΔ*E*〉_0_) – *F*_1_((〈ΔΔ*E*〉_1_) = *F*_1_[*ρ*_0_] – *F*_1_[*ρ*_1_] = –Δ*W* + + 〈ΔΔ*E*〉_2_.

Borgis and coworkers have reported a similar relation to compute the reorganization free energies using MDFT.^39^ Under the assumption that Marcus theory is valid - hence that the two reorganizations free energies are equal, *i.e. λ* = *λ*_0_ = *λ*_1_, eqn (18) and (19) reduce to:20
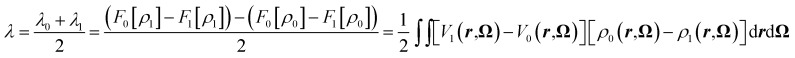
which is equivalent to the linear response formula 
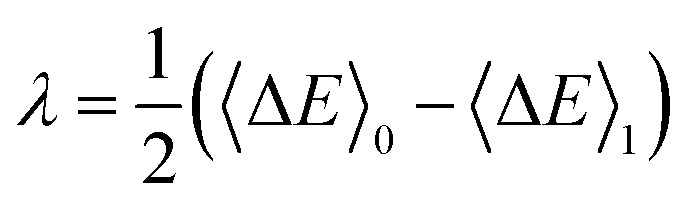
 often used in molecular simulations. We also note that, as for the usual variable Δ*E* in MD, the exact relation introduced by Warshel is satisfied:

21 *F*_1_((〈ΔΔ*E*〉_*η*_) = *F*_0_((〈ΔΔ*E*〉_*η*_) + ) + 〈ΔΔ*E*〉_*η*_.

This is a corollary of eqn (34) with *η* = 1. In the next subsection we explain how the average vertical energy gap, reorganization free energies and free energy curves are computed using MDFT.

#### 1.3.2 Computational details

To study a given system, we shall minimize functionals corresponding to different external potentials *V*_*η*_ according to eqn (13). We consider only cases for which the Lennard-Jones sites of the solute remain unchanged between state 0 and state 1, so that the energy gap reduces to the difference in the electrostatic potential energy of the solute in the field generated by the solvent molecules. This can be computed using the electrostatic potential generated by states 0 and 1, while the vertical energy gap can be computed using eqn (14).

As shown above, the free energies corresponding to these values of the energy gaps are

22 *F*_*A*_((〈ΔΔ*E*〉_*η*_) = *F*_*A*_[*ρ*_*η*_]

with *A* = 0,1. To construct the FEC as in Fig. 1 we first minimize the functional of eqn (2) for several values of *η* to obtain *ρ*_*η*_, next compute the value of the average vertical energy gap, and finally evaluate *F*_0_ and *F*_1_ for the different *ρ*_*η*_.

An alternative route to compute the FEC was previously proposed by Hirata *et al.*^55^,^56^ using another implicit solvent method, RISM. We show in Appendix C that the thermodynamic cycle they propose is equivalent to the present scheme, although not expressed in a free energy density functional language. Now that we have shown how MDFT can be used to investigate ET reactions, the following section is dedicated to assess the validity of this approach on simple and complex solutes.

## 2 Applications

### 2.1 ET between Cl^0^, Cl^+^ and Cl^–^ ions

In this article we focus on the difficult case of aqueous solvation, but calculation for simpler solvents such as acetonitrile or CO_2_ are expected to give results of comparable quality. We apply the necessary correction due to periodic boundary conditions to charged solutes^57^,^58^ and an additional correction accounting for the overestimation of the pressure within HNC^53^ to both neutral and charged solutes.

To allow comparisons, we chose a system which has been extensively studied using MD by Hartnig *et al.*^19^ This model of chlorine consists of one Lennard-Jones site, with *σ* = 4.404 Å and *ε* = 0.4190 kJ mol^–1^, and a charge equal to –1, 0 or 1 elementary charges *e*. To compute the FEC of the atom and the 2 ions with a good accuracy we ran MDFT calculations with a solute charge varying in steps of 0.1 elementary charges. We used a 40 × 40 × 40 Å^3^ box with 120^3^ spatial grid points and 196 possible orientations per spatial point. The solvent is SPC/E water for which the exact direct correlation function projected on a basis of rotational invariants was obtained by Belloni *et al.* using a hybrid Monte Carlo plus integral equation approach.^59^–^61^ All simulations are carried out at 298.15 K.

**Fig. 2:**
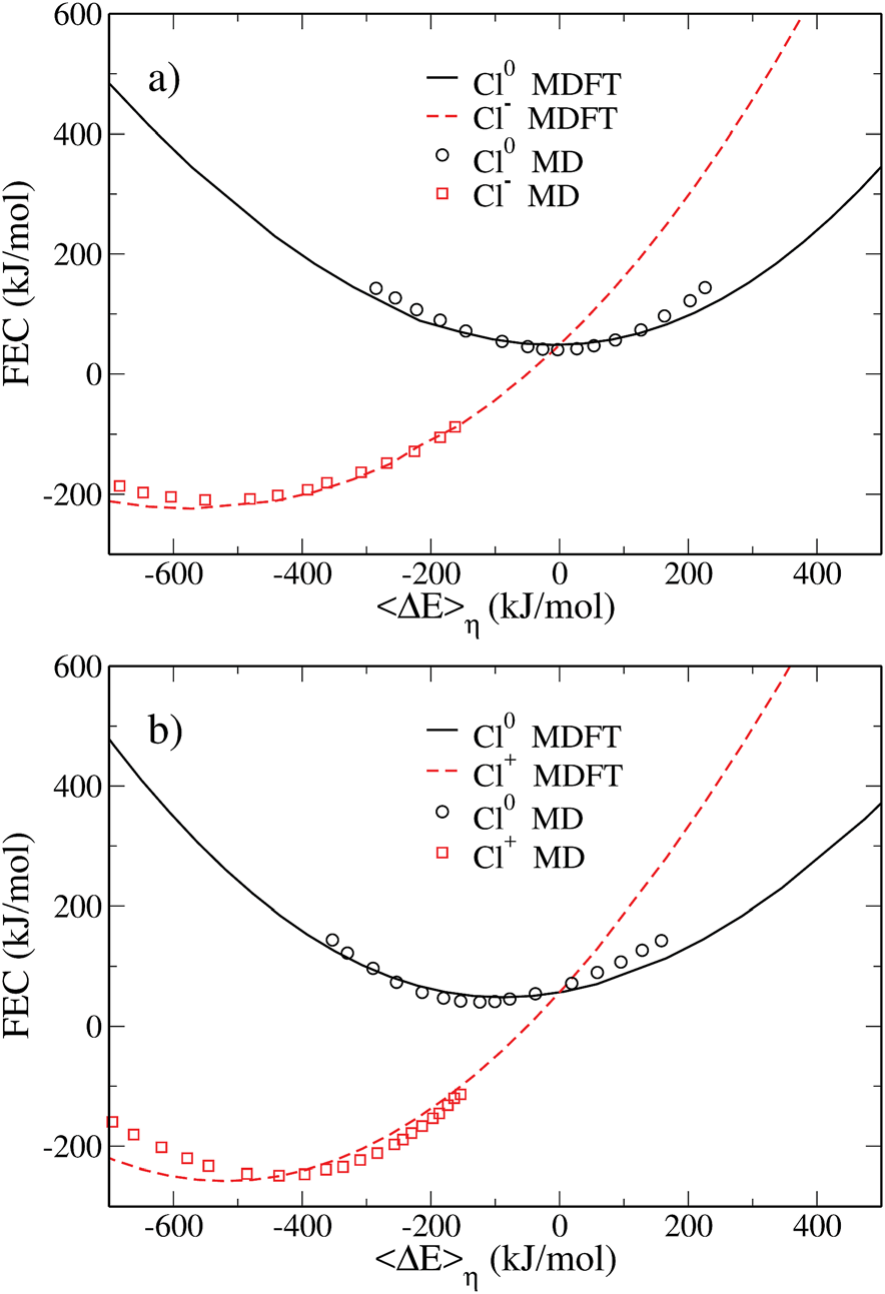
Pairs of free energy curves of (a) Cl^0^/Cl^–^ and (b) Cl^0^/Cl^+^ as a function of the average vertical energy gap. The black solid line and the dashed red line correspond to the MDFT results for the atom and the ions, respectively. Those results are compared to Hartnig's work^19^ reported as a function of the absolute vertical energy gap. The black circles correspond to the atom and the red squares to the ions.

The FEC are shown in Fig. 2 which compares the MDFT results (solid lines) to the MD results^19^ (symbols). The representation adopted here differs from that used by Hartnig and Koper, since we report the FEC as a function of the vertical energy gap and do not apply an arbitrary vertical shift of the curves. The methodology used to plot the MD data in this representation is described in Appendix D. This representation is better suited to highlight some features of the ET. For instance, we note that both pairs of FEC cross when the average free energy gap is equal to 0, as expected.

The agreement between MD and MDFT is satisfactory, the main difference being a shift of the MDFT curves towards negative values of The agreement between MD and MDFT is satisfactory, the main difference being a shift of the MDFT curves towards negative values of 〈ΔΔ*E*〉_*η*_ for the cation. The most interesting observation from Fig. 2 is the consistency between the curvature obtained by MD and by MDFT. In particular, Cl^0^ and Cl^+^ exhibit a similar curvature while that of Cl^–^ is larger, indicating that the neutral to anion ET does not follow the Marcus picture while the neutral to cation ET does. To be more quantitative, we computed the reorganization free energy associated with the three species, based on the curvature by fitting the data within 90 kJ mol^–1^ from the minimum with the following expression23
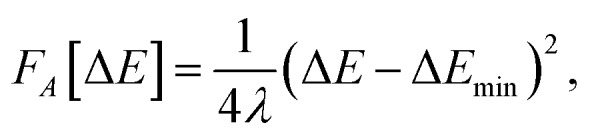
a strategy that was adopted by Hartnig *et al.* This assumes that the curve can be fitted by a parabola. The expression linking the curvature and the parabola parameter in eqn (23) is derived using Marcus theory.

The reorganization free energies obtained *via* MDFT and MD are compared in Table 1. For the MD data, we report the values of the free energies given in the original work, in addition to the one we have recomputed to keep the fitting procedure consistent between the two approaches. MDFT overestimates the reorganization free energies compared to MD. However, comparing the values of the reorganization free energies between species leads to conclusions similar to those concerning the curvature of the FEC. The neutral atom and the cation have a similar reorganization free energy, while the anion has a much larger one.

**Table 1:** Reorganization free energies computed *via*eqn (23) using data below 90 kJ mol^–1^ from Fig. 2. For MD, we recomputed the reorganization free energy using points extracted from Fig. 4 of ref. 19. In parenthesis we report the original data of that publication

Species	*λ*_MDFT_ (kJ mol^–1^)	*λ*_MD_ (kJ mol^–1^)
Cl^0^	233	153 (132)
Cl^–^	297	263 (252)
Cl^+^	216	165 (177)

The simple picture emerging from the comparison of curvatures is however misleading. Table 1 suggests that a single reorganization free energy can be associated with each solute, but this does not hold for several reasons: (i) it assumes that the FEC of a solute is a parabola (ii) it neglects the other solutes involved in the ET. We should refer to the reorganization free energy for a given 0 → 1 ET reaction as defined in Fig. 1, because the meaningful physical quantity is a free energy, not the curvature of a fitting curve. To illustrate this point, we report in Table 2 the reorganization free energies computed using eqn (18) and (19). These free energies for the Cl^0^ → Cl^+^ reaction are almost identical to those reported in Table 1. This is consistent with the Marcus picture: if the two FEC are identical parabolas, there is a unique *λ* parameter defining the curvature and the two free energy differences. However, in the Cl^0^ → Cl^–^ ET reaction the reorganization free energy of state 0 is much larger than in the other ET reaction. This is a consequence of the larger curvature of the second state. The reorganization free energy of Cl^–^ is significantly reduced compared to those listed in Table 1, another consequence of the smaller curvature of state 0.

**Table 2:** Reorganization free energies computed by MDFT *via*eqn (18) and (19) for both ET reactions

Species	Cl^0^/Cl^–^ (kJ mol^–1^)	Cl^0^/Cl^+^ (kJ mol^–1^)
Cl^0^	297	214
Cl^–^	264	N/A
Cl^+^	N/A	218

This difference between the reorganization free energies is a further indication that the 0 → –1 ET does not follow Marcus theory.

**Fig. 3:**
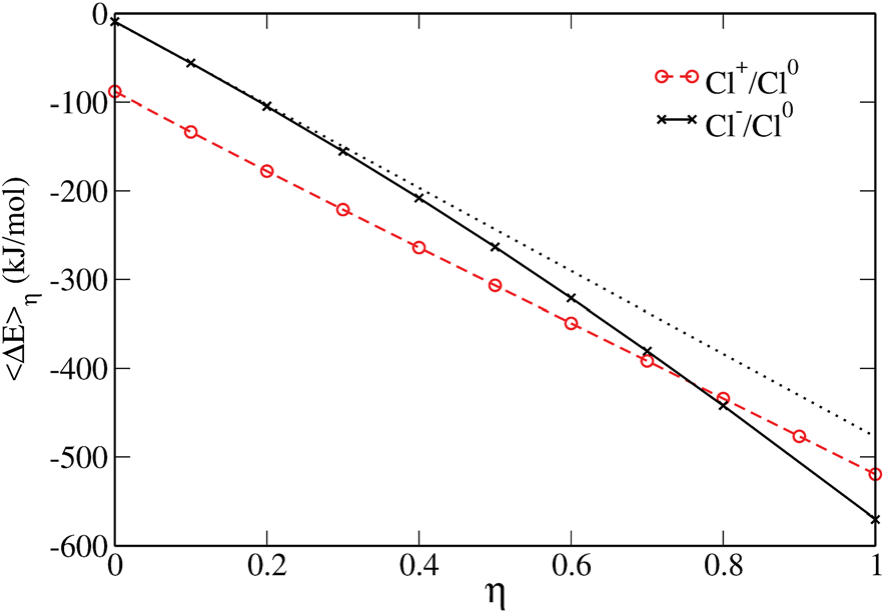
Average value of the vertical energy gap *versus* the coupling parameter *η*. The Cl^0^ → Cl^–^ ET reaction is shown in solid black, the Cl^0^ → Cl^+^ in dashed red. The dotted curve is a linear fit to the first values of vertical energy gap in the case of the ET reaction involving the anion, it is shown as a guide to the eye.

Another way to check if the ET reaction follows the Marcus picture is to consider the evolution of the average vertical energy gap Another way to check if the ET reaction follows the Marcus picture is to consider the evolution of the average vertical energy gap 〈ΔΔ*E*〉_*η*_ with the parameter *η*. As mentioned earlier, such a curve is linear in Marcus theory. The evolution of the average vertical energy gap with *η* is presented in Fig. 3. For the neutral to positive charge transfer, the vertical energy gap does vary linearly with the coupling parameter *η*. In contrast, for the neutral to anion ET a non-linear variation is observed indicating a deviation from Marcus theory.

Thus, the curvatures of the FEC, the values of the reorganization free energies and the variation of Thus, the curvatures of the FEC, the values of the reorganization free energies and the variation of 〈ΔΔ*E*〉_*η*_ with respect to *η* consistently indicate a different behavior for the two ET reactions. This has already been noticed by Hartnig *et al.*, who rationalized this observation by arguing that while the distance between the solute and the oxygen of the first solvation layer remains similar for all oxidation numbers, the hydrogen is much closer to the solute in the case of the anion. This causes a “shrinking” of the first solvation shell in the case of Cl^–^ which differs considerably from the solvation shell of the neutral and positive solutes. Such a difference in the solvation shells of the two species cannot be properly captured by linear response assumed in Marcus theory.

**Fig. 4:**
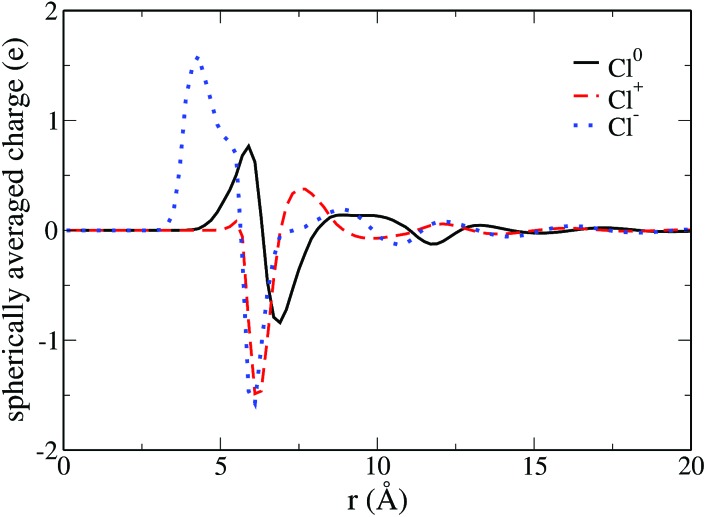
Spherically averaged solvent charge density as a function of the distance to the solute. The curve corresponding to the neutral solute is in solid black, the one of the cation is in dashed red and the one of the anion in dotted blue.

Since MDFT gives access to the solvent density, we can also investigate the solvation structure. We compute the solvent charge density24
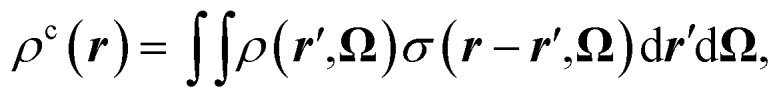
where *σ*(***r***,**Ω**) is the charge distribution at point ***r*** of a single solvent molecule located at the origin, with the orientation **Ω**25
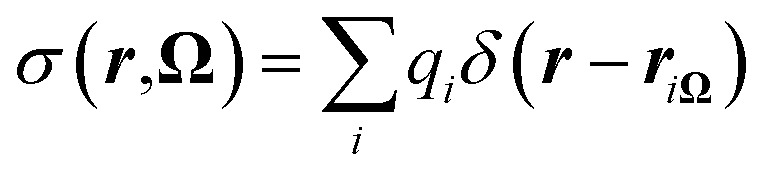
where the sum runs over the solvent sites, *δ* is the Dirac distribution, ***r***_*i***Ω**_ is the position of site *i* and *q*_*i*_ its charge. The spherically averaged one-dimensional solvent charge densities are reported in Fig. 4 for the 3 oxidation numbers as a function of the distance to the solute. For all solutes, we observe a zone of zero charge density for small values of *r*, *i.e.* close to the solute, corresponding to the absence of water molecules. Then, alternating regions of positive and negative charge point to a preferential orientation of the solvent in the solvation shells. Finally, zero charge density is reached far from the solute at large *r* when a bulk behavior without preferential orientation is recovered.

If we first consider the neutral and positive solutes, we observe in Fig. 4 that the preferential orientation of water in the first solvation shell reverses between the neutral solute and the cation. Around the cation, the water molecules in the first solvation shell have their oxygen pointing toward the solute. For the neutral species the hydrogen of water molecules are the closest to the solute. However the positions of the first extrema are similar, 5.9 Å for Cl^0^, 6.3 Å for Cl^+^. This indicates that the two solvation shells essentially differ by the orientation of the water molecules. On the contrary, solvent molecules are much closer to the anion, where the first maximum originating from hydrogen is located at 4.3 Å. There is a shrinking of the first solvation shell for the anion, in agreement with Hartnig *et al.*^19^ It is confirmed by the comparison of the partial molar volume computed thanks to the equilibrium densities, namely 60 Å^3^ for Cl^0^ and Cl^+^ and 6 Å^3^ for Cl^–^. This difference in the solvation shell explains why Marcus theory fails to describe this ET reaction.

### 2.2 Solid/solvent interface

We now turn to the study of the influence of a solid/solvent interface on the ET reaction. There are only few such studies available due to the computational cost of MD which is to date the only simulation tool used in this context. It is worth mentioning the investigations by Remsing *et al.*^62^ who used MD and by Li *et al.*^18^ based on coarse grained MD. In the former, ions are highly confined between two MnO_2_ sheets and confinement is kept constant throughout the study. In the ESI of Li's paper, the authors report the evolution of the reorganization free energy when the ion moves towards graphite sheets. They used umbrella sampling to constrain the position of the redox active site in the direction *z* perpendicular to the surface but no constraint was applied on the lateral coordinates. The dependence of the reorganization free-energy on the distance between solute and electrode was subsequently obtained through binning in the *z*-direction. In this set-up the position of the solute is not frozen but may fluctuate around value of *z* under consideration, taking all possible values in *x* and *y*. The reported reorganization free energy is hence a statistical average.

The computational efficiency of MDFT allows a systematic study of the evolution of the reorganization free energy when the solute carrying the charge approaches an atomistically resolved wall. Because the solute is kept fixed in the MDFT calculation it is not necessary to resort to biasing techniques to constrain its position and no fluctuations blur the reorganization free energies. We consider the Cl^0^ → Cl^+^ ET with the parameters introduced in Section 2.1 and study the influence of the proximity of a wall made up of 400 atoms arranged as the (100) surface of a fcc crystal. The size of the wall is 40 × 40 Å^2^ and the distance between neighbouring atoms is 2 Å. Each atom is modeled by a Lennard-Jones site with parameters *σ* = 3.37 Å and *ε* = 0.23 kJ mol^–1^ similar to that used to model graphite atoms in previous studies.^63^ To study separately the effect of the solvent on the ET we remove direct interactions between the solute and the wall. We used a 40 × 40 × 40 Å^3^ cubic box with 3 grid points per Å and 196 discrete orientations per grid point.

**Fig. 5:**
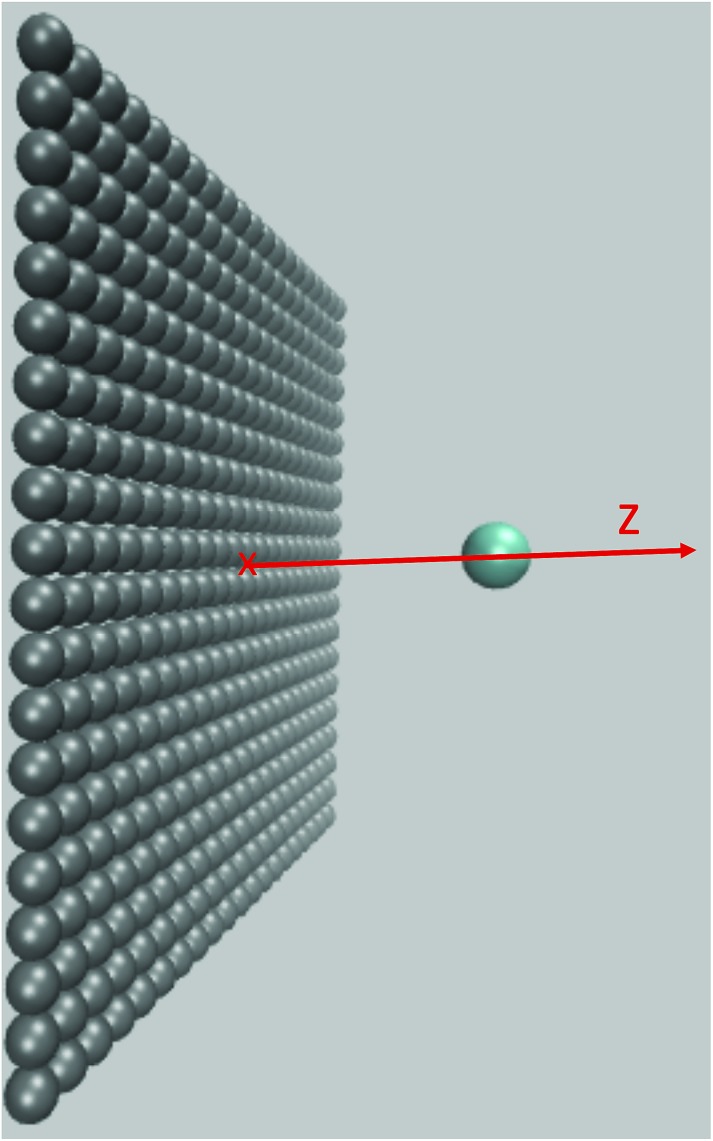
Snapshot of system under consideration: the flat wall is shown in grey, the solute in blue. The solute is moved along the *z* direction perpendicular to the wall.

**Fig. 6:**
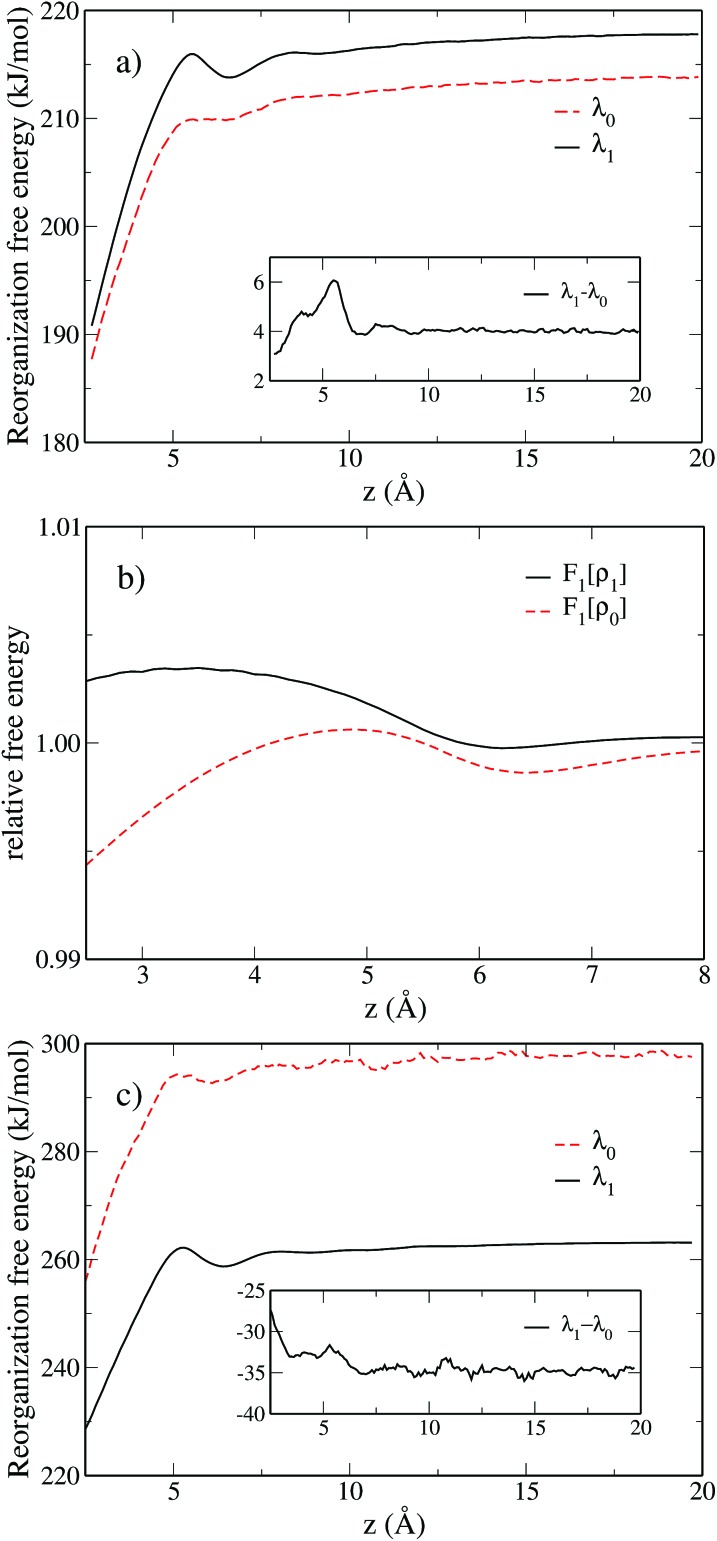
(a) Variation of the reorganization free energy for the Cl^0^ → Cl^+^ ET with the distance between the solute and the wall. The curve corresponding to the neutral state is displayed in dashed red, the one for the charged solute in solid black. The difference between *λ*_1_ and *λ*_0_ is shown in the inset. (b) Zoom on the two components *F*_1_[*ρ*_0_] and *F*_1_[*ρ*_1_] of *λ*_1_ for the Cl^0^ → Cl^+^ ET, the two quantities have been normalized by their bulk value to assist visualization. (c) Same as plot (a) for the Cl^0^ → Cl^–^ ET.

We move the solute along the *z* axis perpendicular to the surface as illustrated in Fig. 5, with 175 calculations from *z* = 2.5 Å to *z* = 20 Å in steps of d*z* = 0.1 Å. The reorganization free energies of the Cl^0^ → Cl^+^ ET computed using eqn (18) are displayed in panel a) of Fig. 6 in solid black for the charged solute and in dashed red for the neutral solute. The two curves are similar and differ by less than 3 kJ mol^–1^. This is a small difference consistent with the result of Li *et al.*^18^ who reported that the ET of an iron atom dissolved in an ionic liquid next to a polarizable planar electrode follows Marcus' scenario.

**Fig. 7:**
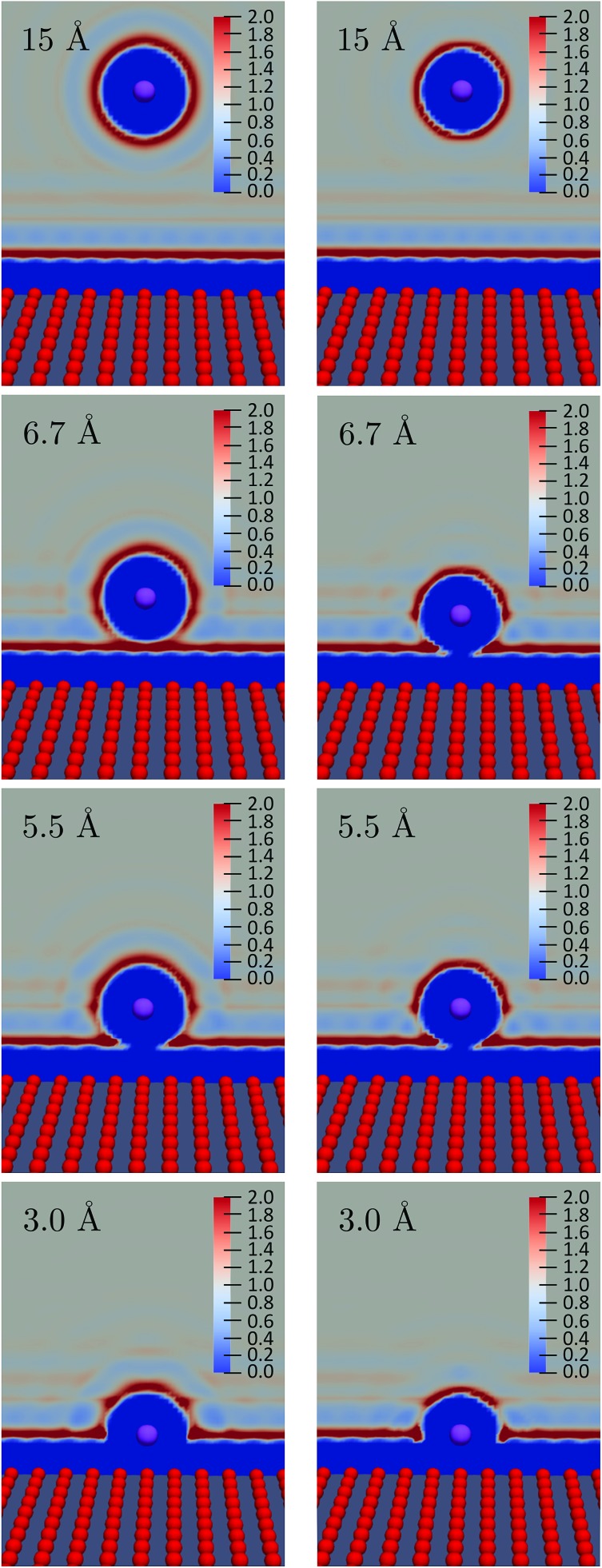
Slices of the solvent density for various values of the distance *z* from the wall. The neutral solute data are shown is in the left-hand column, while the cation data are shown in the right-hand column.

We observe a decrease in the reorganization free energy as the solute approaches the plane. We can rationalize this observation by realizing that the wall truncates the solvation shell around the solute. This effect is illustrated in Fig. 7 which shows slices of the density profile around the neutral (left column) and charged (right column) solutes for different values of *z*. As the solute approaches the wall, there are fewer solvent molecules to rearrange when passing from one equilibrium solvation state to the other. This reduces the cost of the reorganization and explains the decrease of the free energy curves for small *z*. In the limit of total confinement the reorganization free energy would vanish. The upper panel of Fig. 6 shows that reorganization free energy of the charged solute exhibits a maximum around 5.5 Å. We rationalize this effect by decomposing *λ*_1_ into its components *F*_1_[*ρ*_1_] and *F*_1_[*ρ*_0_] in the lower panel of Fig. 6. While *F*_1_[*ρ*_0_] exhibits a marked maximum around 5 Å, *F*_1_[*ρ*_1_] has a maximum around 3.4 Å which is flatter. Their difference consequently gives rise to the oscillatory behavior of *λ*_1_ around 5.5 Å.

The first solvation shell of the neutral solute at 5.5 Å is in contact with the first fluid layer adsorbed on the wall. When the solute gets closer, the solvation shell is reduced. This reduces the unfavorable electrostatic term. It also decreases the cavity term which measures the cost of expelling the solvent from the region around the solute. This explains the maximum of *F*_1_[*ρ*_0_]. Considering the density for the charged solute, the “contact” between the solvation shell of the solute and the fluid layer adsorbed on the wall is also found around 5.5 Å. However, for the cation the truncation of the solvation shell decreases both the favorable electrostatic term and the unfavorable cavity term. This could explain why *F*_1_[*ρ*_1_] is rather flat compared to *F*_0_[*ρ*_1_] and why the position of the maximum is shifted to the left. For *z* > 10 Å, the reorganization free energies reach a plateau corresponding to the bulk value of Table 2.

The inset of panel (a) shows the difference between *λ*_1_ and *λ*_0_ for the for the Cl^0^ → Cl^+^ ET, it presents a maximum at 5.5 Å, *i.e.* where the reorganization free energies also have a maximum. The difference never exceeds 6 kJ mol^–1^, so that Marcus' hypothesis is satisfied at all distances. This is clearly not the case for the Cl^0^ → Cl^–^ ET. Indeed, far from the wall *λ*_0_ and *λ*_1_ differ by 34 kJ mol^–1^ as reported in Table 2. However when the solute approaches the wall, this difference is reduced, indicating a decrease of the deviation the from linear response approximation. Again, this can be rationalized by the truncation of the solvation shell.

**Fig. 8:**
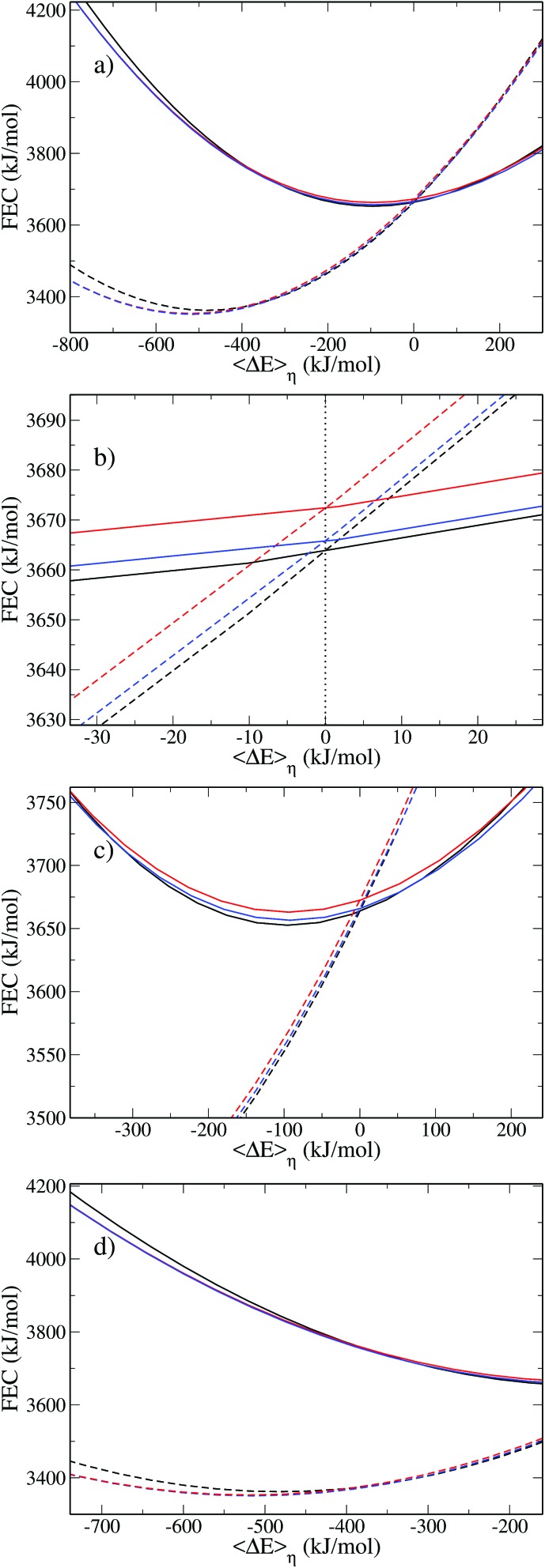
(a) Free energy Cl^0^ (full curve) and Cl^+^ (dashed curve) for various values of *z*. The black curves correspond to *z* = 3.0 Å, the red curves to *z* = 5.5 Å and the blue curves to *z* = 6.7 Å. Panel (b) is a zoom around = 6.7 Å. Panel (b) is a zoom around 〈ΔΔ*E*〉_*η*_ = 0.0 kJ mol^–1^, represented by a dotted lined. The (c) and (d) panels show zooms around the minimum of the atom and cation free energies respectively.

To illustrate the numerical efficiency of the method, we also computed the FEC for various positions: *z* = 3.0 Å is in the region where the reorganization free energy decreases, *z* = 5.5 Å and *z* = 6.7 Å correspond to the first maximum and subsequent local minimum in Fig. 6. The FEC for the atom and cation are presented in Fig. 8. Each pair of curves cross at a point of vanishing vertical energy gap, as expected. When the solute gets close to the wall the minimum of the cation FEC is shifted towards positive values, which is consistent with the above-mentioned truncation of the solvation shell. Finally, the parabolas corresponding to *z* = 3.0 Å are wider than those for higher values of *z*, which is consistent with the smaller value of *λ* close to the wall reported in Fig. 6.

One of the advantages of MDFT is the possibility to split the free energy into entropic, solute–solvent and solvent–solvent contributions according to eqn (2):26
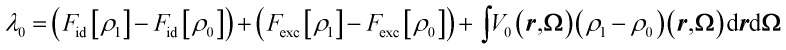

27
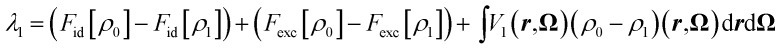



**Fig. 9:**
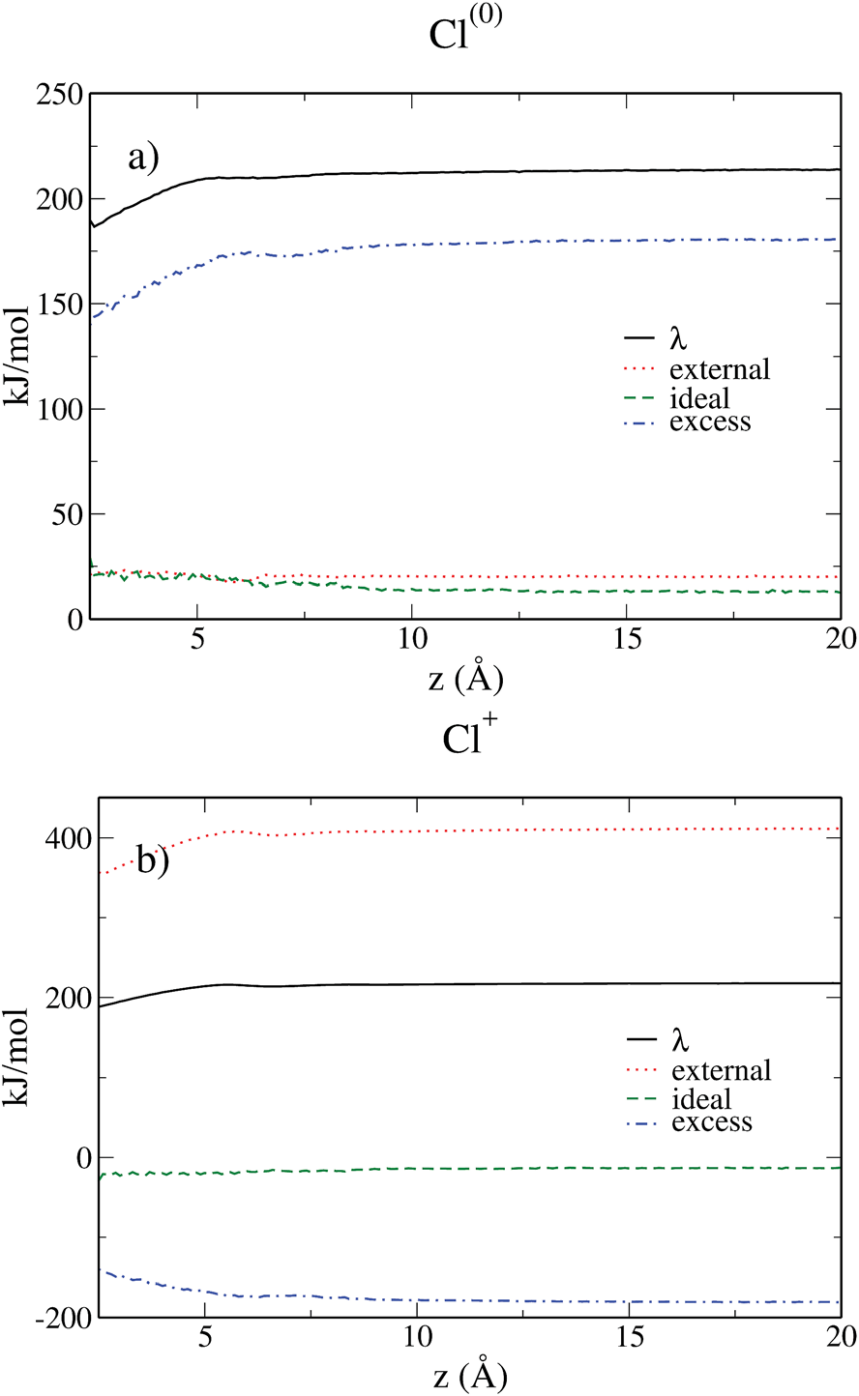
Reorganization free energies and their different contributions for Cl^(0)^ (a) and Cl^+^ (b) as a function of the distance from the wall computed as computed within MDFT. The reorganization free energy is in black, the ideal term is in green, the excess term in blue and the external term in red.

Fig. 9 shows the various contributions to the reorganization free energy for the neutral and the charged solutes. To our knowledge, this is the first time that such a decomposition of the reorganization free energy is reported.

A first conclusion emerging from eqn (3), (6), (26) and (27) is that the ideal and excess contributions are exactly opposite for the neutral and the charged solutes. For both solutes, the ideal term due to the entropic contribution remains quite small and hardly varies with the distance from the electrode.

For the neutral solute the external contribution is small due to the absence of electrostatic interactions and more than 80% of the reorganization free energy is due to the excess term, *i.e.* the solvent–solvent contribution. In contrast, for the charged solute the main contribution is due to the electrostatic interaction between the solute and the solvent, which is roughly twice in absolute value than the solvent–solvent term. Even if we already know from the previous subsection that the Cl^0^ → Cl^+^ transfer does satisfy the linear response approximation, *i.e. λ*_0_ = *λ*_1_, it is fascinating to observe the compensation of the three contributing terms resulting in this equality. When the solutes approaches the wall, the linear response approximation gets even better as evidenced in Fig. 6 where the curves of *λ*_0_ and *λ*_1_ converge. This study also illustrates the interest of MDFT not only to compute the relevant free energies, but also to understand the various contributions to the free energy.

## 3 Conclusion

Marcus theory plays a crucial role in the study of ET reactions. This explains why its validity has been investigated extensively using molecular dynamics simulation. However, MD remains computationally very demanding, and has so far been essentially limited to simple systems. Molecular density functional theory has been proposed as an alternative to study solvation because it is computationally much faster, while retaining a molecular description of the solvent. In the present paper, we develop tools to use MDFT to study electron transfer reactions in water using MDFT. We have first derived how to compute the relevant reaction coordinate: the average vertical energy gap. We have also shown how to compute the free energy curves and the reorganization free energies.

We examined the validity of the approach by studying simple solutes, namely the ET reactions between Cl^0^, Cl^–^ and Cl^+^ modeled by a single Lennard Jones site and a point charge. We found a good agreement between the results obtained by MDFT and corresponding MD simulations. We confirmed the effect reported by Hartnig *et al.*, that the ET between neutral and positive solutes is well described by Marcus theory, but not in the case of the transfer between the neutral atom and the anion.

We finally illustrated the potentiality of the method by tackling a more challenging system. We investigated the effect of the presence of a solid/solvent interface on the reorganization free energy, using a model system composed of an atomistically resolved neutral wall which is approached by the solute along the axis perpendicular to the wall. We computed the reorganization free energy for both neutral and charged states and found that they exhibit similar features. The reorganization free energy remains constant when the solute is far from the wall. As it approaches the wall, it exhibits oscillations before decreasing. We rationalized this behavior by considering the evolution of the solvation shell: close to the wall, there is less solvent to reorganize in the first solvation shell, thereby reducing the free energy cost.

This work is a first step towards the study of ET reaction in water and at electrode/water interfaces based on MDFT. The solvent effect sometimes called outer-sphere contribution is not the only mechanism playing a role in the ET reaction. The rearrangement of the electron cloud of the solute entering the so-called inner-sphere contribution may also play an important role. This effect is well taken into account in QM/MM calculation. There are mainly two approaches to deal with the MM part in such calculations. The first one is to use MD, which takes into account the molecular nature of the solvent, but remains computationally costly. The second one is to use PCM-like models in which the solvent is described as a dielectric continuum. This approach neglects the molecular nature of solvent. As a consequence, it always assumes the validity of the linear response approximation and cannot properly describe systems violating Marcus theory. The strength of this method is its numerical efficiency: calculations are almost instantaneous. MDFT is thus a promising alternative to those two approaches to account for solvation in QM calculations: even if it is computationally more demanding than PCM its computational cost remains negligible compared to the cost of the QM calculation while its accuracy is comparable to MD. To that end, we are currently working on coupling MDFT with electronic structure calculations such as electronic density functional theory. We also wish to develop a framework allowing for the description of the polarizability of the wall to describe electrodes at fixed electrode potential and study electrochemical reactions. These two objective are currently under investigation, in a attempt to develop a computationally efficient MDFT toolbox to tackle ET reactions.

## A Proof that there is a one-to-one mapping between *ρ*_*η*_ and and 〈ΔΔ*E*〉_*η*_

A straightforward consequence of eqn (14) is that the average vertical energy gap is uniquely defined by the density field. Following Mermin and Evans,^34^,^35^ we proceed by reductio ad absurdum to show that the average vertical energy gap uniquely determines the external potential and thus the density. Let us assume there exist two potentials *V*_*η*_ and *V*_*η*′_ with *η* ≠ *η*′ giving rise to the same gap *i.e.* 〈ΔΔ*E*〉_*η*′_ = = 〈ΔΔ*E*〉_*η*_. From the expression of the probability distribution in eqn (10) and as stated in Appendix 1 of Evans's article,^35^*V*_*η*_ ≠ *V*_*η*′_ implies *f*_*η*_ ≠ *f*_*η*′_. From the variational principle of the grand potential we have28
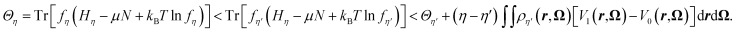



By inverting the primed and unprimed quantities we get29
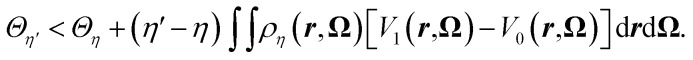



If we now sum eqn (28) and (29) we arrive at30
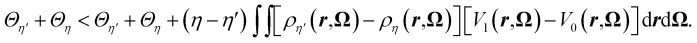



The integral on the r.h.s of eqn (30) vanishes as a consequence of eqn (14) and the assumption that and the assumption that 〈ΔΔ*E*〉_*η*′_ = = 〈ΔΔ*E*〉_*η*_ leading to a contradiction. Consequently, for this family of external potential *V*_*η*_ there is a unique there is a unique 〈ΔΔ*E*〉_*η*_ which corresponds to a given probability distribution *f*_*η*_. We hence have a one to one mapping between all the following quantities

31 *η* ↔ *V*_*η*_ ↔ *f*_*η*_ ↔ *ρ*_*η*_ ↔ ↔ 〈ΔΔ*E*〉_*η*_

where ↔ denotes a one-to-one mapping. The bijections between the three quantities *V*, *f* and *ρ* are always true in the cDFT formalism^35^ while the one involving *η* and *V*_*η*_ is true within the class of potentials we have chosen.

Because there is a bijection between Because there is a bijection between 〈ΔΔ*E*〉_*η*_ and a probability distribution, then the free energy of any state uniquely depends on and a probability distribution, then the free energy of any state uniquely depends on 〈ΔΔ*E*〉_*η*_. To express the free energy as a function of . To express the free energy as a function of 〈ΔΔ*E*〉_*η*_, it is sufficient to take advantage of the one to one mapping between *ρ*_*η*_ and and 〈ΔΔ*E*〉_*η*_, to obtain the expression of eqn (15).

It is worth noticing that we can actually define *F*_0_((〈ΔΔ*E*〉_*η*_) as the Legendre transform of *Θ*_*η*_ with respect to *η*, as , as 〈ΔΔ*E*〉_*η*_ is the conjugate variable of *η*:32
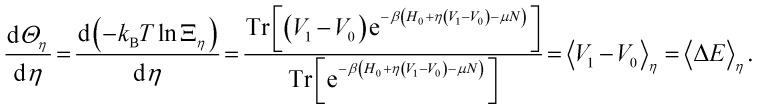



Moreover,33
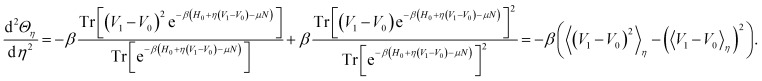



Therefore the second derivative of *Θ*_*η*_ is negative, so that *Θ*_*η*_ is convex and its Legendre transform exists. We can hence define the Legendre transform of *F*_*η*_ by34
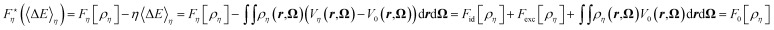
where we split *F*_*η*_ into the sum of its three components as in eqn (2) and use the expression of *V*_*η*_ in eqn (13). While the linear parametrization of the external potential is the only one allowing to define *F*_0_((〈ΔΔ*E*〉_*η*_) as a Legendre transform, any parametrization leads to the same expression for *F*_0_((〈ΔΔ*E*〉_*η*_) and to the same FEC as demonstrated in Appendix B.

## B Two different parameterizations of *V*_*η*_ lead to the same *F*((〈ΔΔ*E*〉_*η*_)

Let us consider the general parametrization for the interpolating potential,

35 *Vsη* = *V*_0_ + *s*(*η*)(*V*_1_ – *V*_0_)

where *s* is a strictly increasing continuous function with *s*(0) = 0 and *s*(1) = 1. We first show that any parametrization verifies the properties demonstrated in Appendix A. For any function *s*, let *γ*,*δ* ∈ [0,1] such as ∈ [0,1] such as 〈ΔΔ*E*〉*sγ* = = 〈ΔΔ*E*〉*sδ*. Using an argument identical to eqn (28) we obtain36
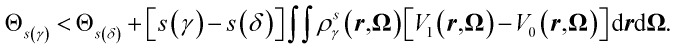



Again the *δ* and *γ* indexes can be interchanged to show a one-to-one mapping between a value of the coupling parameter, the external potential, the equilibrium probability distribution and the equilibrium density. However this mapping now depends on the chosen parametrization *s*,37
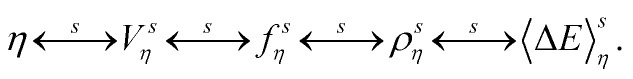



The mapping between The mapping between 〈ΔΔ*E*〉*sη* and *ρsη* leads to:

38 *F*_0_((〈ΔΔ*E*〉*sη*) = *F*_0_[*ρsη*].

This relation does not depend on the choice of the parametrization *s*, but the values of , but the values of 〈ΔΔ*E*〉*sη* and *F*_0_[*ρsη*] do. We now show that any parametrization yields the same FEC.

Let us consider a strictly increasing continuous function with *s*(0) = 0 and *s*(1) = 1. The intermediate value theorem guarantees that *s* takes all the value between 0 and 1, once only. This is true for all *s* and in particular for the identity function corresponding to the linear parametrization. This last property implies that for all *s*(*η*) ∈ [0,1], there exists a unique *α* ∈ [0,1] such that*s*(*η*) = *αVsη* = *V*_*α*_

Since the potential uniquely defines the functional this implies the same equality between all the properties in eqn (37), *i.e.fsη* = *f*_*α*_*ρsη* = *ρ*_*α*_〈ΔΔ*E*〉*sη* = = 〈ΔΔ*E*〉_*α*_*F*_0_[[〈ΔΔ*E*〉*sη*] = *F*_0_[[〈ΔΔ*E*〉_*α*_]

This completes the proof that any parametrization of the intermediate potential leads to the same FEC.

## C Thermodynamic cycle proposed by Chong and Hirata

**Fig. 10:**
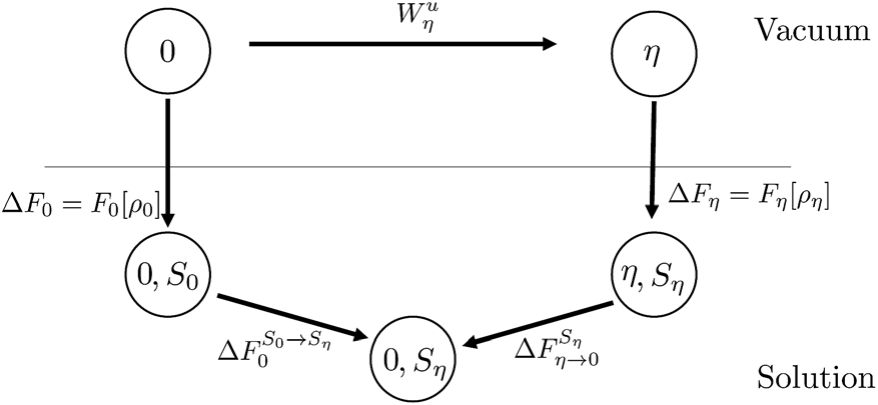
Schematic representation of the thermodynamic cycle used to recover eqn (40). 0 corresponds to the state under consideration, while *η* corresponds to a fictitious solute which interacts with the solvent *via* an external potential *V*_*η*_. The solvation states in equilibrium with states 0 and *η* are respectively denoted by *S*_0_ and *S*_*η*_. To compute the FEC we need to compute 
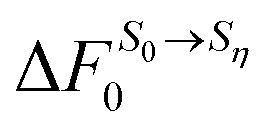
, the free energy cost to modify the solvent configuration around state 0 from *S*_0_ to *S*_*η*_.

Chong and Hirata proposed the thermodynamic cycle displayed in Fig. 10 where 0 and *η* are solutes corresponding to external potentials *V*_0_ and *V*_*η*_ in eqn (13).^55^ The objective is to find the free energy cost to modify the equilibrium solvent configuration around 0 into a solvent configuration which would be in equilibrium with *η*, a quantity denoted by 
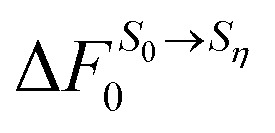
. Starting from 0 in vacuum, it is transformed into *η* spending a work *Wuη*. Then, *η* is solvated in its equilibrium solvent configuration *S*_*η*_. This step corresponds to the solvation free energy Δ*F*_*η*_. The fictitious solute is transformed into 0 while the solvent configuration is frozen within *S*_*η*_. The free energy cost of this step 
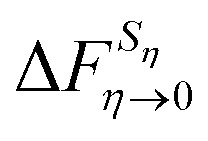
 can be split into the sum of two terms. The first one is the reversible work to transform the solute in vacuum: it is the opposite of *Wuη*. The second term is the work *Wvη* to transform the solute against the field created by the solvent configuration *S*_*η*_ which can be expressed using our previous notation as:

39 *Wvη* = = 〈*V*_0_ – *V*_*η*_〉_*η*_.

The final quantity required to close the cycle is the free energy cost to solvate 0 into its equilibrium solvent configuration. This corresponds to the solvation free energy of state 0, Δ*F*_0_. By closing the cycle, Chong and Hirata obtain the following formula:40
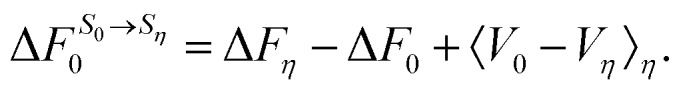



If we replace the solvation free energy by the functional of the present work, eqn (40) becomes41
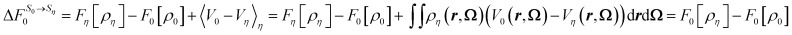
which is equivalent to our previous finding.

## D Modification of Hartnig and Koper's data to plot Fig. 2

In their paper, Hartnig and Koper represented their free energy curves as functions of a generalized order parameter defined as the electrostatic interaction energy between a negative point charge at the site of the solute and the solvent molecules.^19^ Because they only considered solutes with a single Lennard-Jones site which is kept unchanged during the ET the vertical energy gap is equal to their order parameter for an anion and to its opposite for a cation.

They do not mention the use of any finite size effect corrections while we use that proposed by Hünenberger *et al.*^57^,^58^ which applies to our case but also to MD simulations with Ewald electrostatics.^57^ To convert their order parameter in vertical energy gap we thus (i) multiply it by –1 in the case of the cation, (ii) apply the above mentioned electrostatic corrections with the box length parameter of *L* = 24.83 Å reported in their paper.

Finally, Hartnig and Koper shifted all the FEC such that the minimum of each curve is equal to 0. As a consequence their curves do not cross for Finally, Hartnig and Koper shifted all the FEC such that the minimum of each curve is equal to 0. As a consequence their curves do not cross for 〈ΔΔ*E*〉_*η*_ = 0, as should be the case by definition. Because they did not report the values of the solvation free energy, or equivalently the values of the shift applied to each curve, we decided to freely shift vertically the curves corresponding to the atom in order to have the minimum of the curves agree with the value predicted by MDFT. The MD curve for the ion has been subsequently shifted vertically to fulfill the zero gap condition.

## Conflicts of interest

There are no conflicts to declare.
